# Estrogen exposure overrides the masculinizing effect of elevated temperature by a downregulation of the key genes implicated in sexual differentiation in a fish with mixed genetic and environmental sex determination

**DOI:** 10.1186/s12864-017-4345-7

**Published:** 2017-12-18

**Authors:** Noelia Díaz, Francesc Piferrer

**Affiliations:** 1 Institut de Ciències del Mar, Consejo Superior de Investigaciones Científicas (CSIC), Passeig Marítim, 37–49, E-08003 Barcelona, Spain; 2 Present address: Max Planck Institute for Molecular Biomedicine, Röntgenstraße 20, 48149 Münster, Germany

**Keywords:** Climate change, Ecotoxicology, Estradiol, Fish, Phenotypic plasticity, Sex ratio, Sex differentiation, Temperature increase, Transcriptomics

## Abstract

**Background:**

Understanding the consequences of thermal and chemical variations in aquatic habitats is of importance in a scenario of global change. In ecology, the sex ratio is a major population demographic parameter. So far, research that measured environmental perturbations on fish sex ratios has usually involved a few model species with a strong genetic basis of sex determination, and focused on the study of juvenile or adult gonads. However, the underlying mechanisms at the time of gender commitment are poorly understood. In an effort to elucidate the mechanisms driving sex differentiation, here we used the European sea bass, a fish species where genetics and environment (temperature) contribute equally to sex determination.

**Results:**

Here, we analyzed the transcriptome of developing gonads experiencing either testis or ovarian differentiation as a result of thermal and/or exogenous estrogen influences. These external insults elicited different responses. Thus, while elevated temperature masculinized genetic females, estrogen exposure was able to override thermal effects and resulted in an all-female population. A total of 383 genes were differentially expressed, with an overall downregulation in the expression of genes involved in both in testicular and ovarian differentiation when fish were exposed to Estradiol-17ß through a shutdown of the first steps of steroidogenesis. However, once the female phenotype was imposed, gonads could continue their normal development, even taking into account that some of the resulting females were fish that otherwise would have developed as males.

**Conclusions:**

The data on the underlying mechanisms operating at the molecular level presented here contribute to a better understanding of the sex ratio response of fish species subjected to a combination of two of the most common environmental perturbations and can have implications in future conservational policies.

## Background

The sex ratio is an essential demographic parameter in population ecology, and its proper establishment is crucial for the perpetuation of all sexually-reproducing species [1]. In fish, the establishment of the primary sex ratio mostly depends on the genetic and environmental contribution to the process of sex determination and differentiation [2, 3], although other factors such as differential survival can also have an influence.

While downstream genes implicated in gonadal sex differentiation are conserved [4, 5], master sex-determining genes are not [6]. Importantly, and also in contrast to mammals, in all non-mammalian vertebrates estrogens are essential for proper ovarian differentiation [7]. Therefore, blockade of gonadal aromatase, the steroidogenic enzyme that irreversibly converts androgens into estrogens such as estradiol-17ß (E_2_) results in the masculinization of the gonads of genetic females in different species [8–11]. Conversely, exposure to E_2_ feminizes the gonads of genotypic males in many species [9, 12–15].

Temperature increases related to global change and pollution of water bodies, both ultimately due to human activity, greatly influence aquatic ecosystems, with opposing effects on sex ratios of many fish populations. In sensitive fish species, the sex ratio (gonadal differentiation) response to elevated temperatures is an increase in the number of males [16]. Thus, abnormally elevated temperatures often result in a severely male-biased population [17]. On the other hand, many chemicals present in the aquatic environment have a feminizing effect since they are able to disrupt the endocrine system by mimicking the effects of estrogens through binding to the estrogen receptor [18]. Consequently, even at low environmental concentrations, a sufficiently long exposure can lead to the feminization of the entire population [19].

Fish transcriptomes have been analyzed during sexual differentiation [20–25] and after E_2_ exposure [18, 26–28]. Tissue- and gender-specific responses, [29, 30] as well as biogeographical differences [31], have been shown. However, it is still not clear whether the exposure to exogenous steroids elicits changes similar to those occurring during natural physiological processes [32–34]. These exogenous steroids inhibit the expression of several steroidogenic enzymes, as observed in different species [34–37] and thus alter normal hormonal functions [38, 39]. However, most of the studies referred to above were conducted in species with a strong sexual determining system (XX/XY or ZW/ZZ) where sex is highly canalized and not easily influenced by environmental perturbations. This contrast with species with a polygenic sex determination system, where the final sex depends on a delicate balance between endogenous and external stimuli [24].

The European sea bass (*Dicentrarchus labrax*) is a gonochoristic species that lacks sex chromosomes and for which a polygenic system of sex determination involving a two-biallelic system has been proposed [40]. Furthermore, sex determination and differentiation are influenced by environment during early development [41], when temperatures just a few degrees above 17 °C applied during the thermosensitive period (0–60 days post hatch; dph [24, 42]) masculinize about half of the fish that under natural temperatures would develop as females. This masculinization is induced through the hypermethylation of the *cyp19a1a* promoter in females that prevents the synthesis of the E_2_ necessary for ovarian development [43]. It is also known that E_2_ administered during the hormone-sensitive period (HSP = 90–160 days post hatch [44]) can result in feminization of the whole population [14]. The study of European sea bass responses to environmental cues is also interesting because the nursing of this species takes place in coastal shallow waters of 10 m depth [45], that are more sensitive to thermal fluctuations as the ones predicted in current climate change models [46], and also likely containing xenoestrogens that can act as endocrine disruptors [47].

The goal of our study was to compare patterns of gene expression in a species with polygenic sex determination such as the European sea bass at the time when gonads were experiencing opposite pathways of differentiation as a consequence of the environmental cues to which they were exposed. For that we generated two European sea bass populations: 1) one male-biased (78% males) through exposure to elevated water temperature, and another female-biased (100% females) by exposing fish to E2 during the HSP. We then examined gene expression in gonads at 170 dph, i.e., during the sex differentiation period by a custom-made oligo microarray.

## Methods

### Rearing conditions, experimental design and basic data collection

Twenty-four hours post hatch European sea bass larvae from a commercial hatchery (St. Pere Pescador, Girona, Spain) were transferred to the Institute of Marine Sciences “Experimental Aquarium Facility” (ZAE). Larvae densities per tank, environmental and rearing conditions followed the protocols previously described [48]. Fish used for this article were reared and sacrificed in agreement with the European Convention for the Protection of Animals used in scientific experimentation (EST Nu 123, 01/01/91).

The male fish used in this study were siblings of males used in a previous study [24]. Briefly, fish were divided into four tanks and maintained at 17 °C during the first 20 dph. Then, water temperature was raised until 21 °C for two of the groups (Control “HT” and Estradiol “HT-E_2_”) for the remaining two groups; temperature was decreased to 15 °C (Control low temperature “LT” and Estradiol “LT-E_2_”), at a rate of 0.5 °C/day. At ~220 dph (fall) water was left to follow the natural fluctuations in temperature. From 90 to 154 dph, the HT and LT groups (*n* = 150 fish/group) were fed ad libitum two times a day with dry food sprayed with 96% Ethanol, while the other groups E_2_ groups (*n* = 150 fish/group) were fed with the same diet supplemented with Estradiol diluted in 96% Ethanol at 10 mg/Kg food (Additional file 1: Figure S3). No treatment-related mortality was observed.

Following the protocols already detailed in [24]; biometric data (standard length: SL, body weight: BW) was collected periodically and gonads samples for microarray and qPCR analysis of gene expression were taken at 170 dph and immediately frozen in liquid Nitrogen. At 170 dph gonads were fixed in 4% Paraformaldehyde to assess the female stages of oocyte maturation and male spermatogenesis progression [49]. Gonadosomatic (GSI), hepatosomatic (HSI) and carcass (CI) indices were determined to analyze the possible effects of temperature and hormonal exposure on fish maturation at 337 dph.

### RNA extraction and cDNA synthesis

As previously described in [50], total RNA was obtained from 170 dph sea bass gonads using Trizol and a chloroform-isopropanol-ethanol protocol. RNA concentration was measured with a ND-1000 spectrophotometer, and quality was confirmed by examination on 1% agarose/formaldehyde gels. Total RNA was treated with RNase-free DNase, reverse transcribed to cDNA and checked using a Bioanalyzer 6000 Nano LabChip. Samples with a 100–200 ng/μl concentration and RIN values >7 were selected for microarray hybridizations.

### Quantitative real-time PCR (qPCR)

As previously described in [24] qPCR was used to: 1) select high *cyp19a1a* expressors (presumably females) at 170 dph for microarray analysis (Additional file 2: Figure S7) and, 2) validate microarray results and check several genes related to sex differentiation (Additional file 3: Table S1 for a gene glossary). cDNA was always diluted 1:10 for target genes and 1:500 for the *r18S* housekeeping gene (previously validated in [47]). Briefly, primer design and quality checking was done using Primer 3 Plus, primer specificity and performance was checked with a melting curve analysis after amplification (Additional file 4: Table S2: *E*: efficiency between 1.99 and 2.27; slope ranging from −2.6 to −3.3 and *R* ^2^: linear correlations higher than 0.94) and a standard qPCR program was performed. Samples were run in triplicate on an ABI 7900HT in 384-wells plates in a final volume of 10 μl per well with negative controls lacking cDNA/primers always included in duplicate. Data were collected and analyzed using SDS 2.3 and RQ Manager 1.2 software. Primer *E* was used to adjust Ct values and the *r18S* housekeeping gene was used to correct for intra- and inter-assay variations [51].

### Microarray

Five individuals per group were individually hybridized and randomly distributed on different slides to avoid batch effects. Microarrays were hybridized at the Institute of Biotechnology and Biomedicine (UAB, Barcelona). Briefly, RNA was Cy3-labeled with Agilent’s One-Color Microarray-Based Gene Expression Analysis, along with Agilent’s One-Color RNA SpikeIn Kit), cRNA was purified, quantified on a ND-1000 Nanodrop, verified on a Bionalyazer 2100, hybridized in a custom sea bass array (Agilent ID 023790), washed and scanned (see detailed protocol in [24]). Intensities and control features were checked by Agilent’s Feature Extraction software version 10.4.0.0. The platform that validates the array can be seen at Gene Expression Omnibus (GEO)-NCBI database (GPL13443). Datasets used in this article are accessible at GSE52307 for the LT and HT samples and at GSE52938 for LT-E_2_ and HT-E_2_ ones.

### Statistical analysis of data

Briefly, data were checked for normality (Kolmogorov-Smirnov’s test), homoscedasticity of variance (Levene’s test) and log-transformed when needed. GSI, HSI and CI data were arcsine transformed before any statistical analysis. A two-step cluster analysis previously validated and described elsewhere [52] of 2DCt *cyp19a1a* values at 170 dph was used to select the highest *cyp19a1a* expressors per sample for the hybridizations based on the available expression data (Additional file 2: Figure S7). One-way analysis of variance (ANOVA) was used to determine differences between relative *cyp19a1a* mRNA levels resulting from qPCR high and low expressors and for length, weight, GSI, HSI and CI data sets. Post hoc multiple comparisons (Tukey’s HSD test) were done when statistical differences were present. Data are expressed as mean ± SEM (standard error of the mean). In this study, differences were accepted as significant when *P* < 0.05. Chi-square test with Yates’ correction [53] was used for sex ratio analysis and qPCR 2DCt values [51] were analyzed by Student’s *t*-test. Analyses were performed using IBM SPSS Statistics 19.

Briefly, microarray raw data from the Feature Extraction output files was corrected for background noise [54] and quantile normalized [55]. Limma [56] was used to analyze differential expression, and then corrected for multiple testing (False Discovery Rate method, FDR). Genes were considered to be differentially expressed genes (DEG) when the absolute fold change between the two compared groups, was higher than 1.5, the adjusted *P*-value was lower than 0.05 and were reliable in all samples. A Principal Component Analysis was performed to visualize the variability of the samples (Additional file 5: Figure S4). Statistical analysis was performed with the Bioconductor project (http://www.bioconductor.org/ ) in the R statistical environment (http://cran.rproject.org/) [57].

### Gene annotation enrichment analysis

Briefly, Genecards (http://www.genecards.org/) and Uniprot (http://www.uniprot.org) were used to assign gene names, gene symbols, synonyms and functions. The web based tool AMIGO (http://amigo.geneontology.org/cgi-bin/amigo/go.cgi; [58]) was used to obtain sequences of the DEG, Blast2GO software [59], KEGG (http://www.genome.jp/kegg/) and DAVID (http://david.abcc.ncifcrf.gov); [60, 61]) were used to assign GO terms as well as the pathways associated to these genes. In addition, Blast2GO was used with a reference set containing all the genes from the custom-made microarray to check GO term results by a two-tailed Fisher’s Exact Test with Multiple Testing Correction of FDR [62]. Physical and functional protein interactions of the genes were modeled with a web based tool STRING v9.1 (http://string-db.org/); [63]) using the STRING human database as a background.

## Results

### Biometries

Standard length (SL) and body weight (BW) were assessed at 170 (when samples for microarray hybridizations and qPCR were taken) and 337 dph (when sex ratio was assessed). At 170 dph, in both the low and the high *cyp19a1a* expressors, the Estradiol (E_2_) treated fish were shorter (*P* < 0.01) and lighter (*P* < 0.001) than those of the high temperature (HT) group indicating a negative effect of E_2_ on growth. These differences were also present when comparing low temperature (LT) vs. low temperature plus Estradiol (LT-E_2_) fish (Table 1). At 337 dph, sexual growth dimorphism (SGD) was not observed in the HT group, since there were no differences in BW between sexes (Table 2). In contrast to the situation observed at 170 dph, at 337 dph HT females, despite being slightly bigger and heavier than E_2_-exposed females, showed no differences for SL nor BW (Table 2) and neither for hepatosomatic (HIS) nor carcass index (CI) (data not shown).

**Table 1:** Growth of European sea bass juveniles at 170 days post hatch according to treatment and *cyp19a1a* expression levels by qPCR

Low *cyp19a1a* expressors				High *cyp19a1a* expressors		
Treatment	N	Length (cm)	Weight (g)	N	Length (cm)	Weight (g)
LT	10	9.25 ± 0.196^b^	13.16 ± 0.967^b^	6	9.33 ± 0.061^b^	13.53 ± 0.581^b^
HT	9	9.86 ± 0.109^a^	17.41 ± 0.877^a^	7	10.28 ± 0.495^a^	19.35 ± 2.955^a^
LT-E_2_	16	9.15 ± 0.071^b^	12.70 ± 0.385^b^	4	9.20 ± 0.141^b^	12.72 ± 1.130^b^
HT-E_2_	12	9.40 ± 0.176^ab^	13.75 ± 0.875^b^	8	9.49 ± 0.193^ab^	14.65 ± 0.862^ab^

One- way ANOVAs for length and weight comparing low *cyp19a1a* expressors for the four treated groups as well as for the high *cyp19a1a* expressors. Results are shown as mean ± SEM. Different letters indicate statistical differences (*P* < 0.05) between groups

**Table 2:** Growth of European sea bass juveniles at 337 days post hatch according to treatment and sex

Females				Males		
Treatment	N	Length (cm) ± SEM	Weight (g) ± SEM	N	Length (cm) ± SEM	Weight (g) ± SEM
LT	40	12,45 ± 0,180^a^	33,16 ± 1560^a^	26	11,66 ± 0,223^b^	27,22 ± 1867^b^
HT	16	12,22 ± 0,171^a^	33,88 ± 3044^a^	60	12,36 ± 0,171^ab^	33,79 ± 1544^b^
LT-E_2_	79	12,28 ± 0,138^a^	31,76 ± 1247^a^	4	13,55 ± 1582^a^	48,5 ± 20,963^a^
HT-E_2_	41	11,82 ± 0,236^a^	29.45 ± 2.160^a^	0	–	–

Results are shown as mean ± SEM. Different letters mark statistical differences (*P* < 0.05) between groups

### Sex ratio and gonadosomatic index (GSI)

**Fig. 1:**
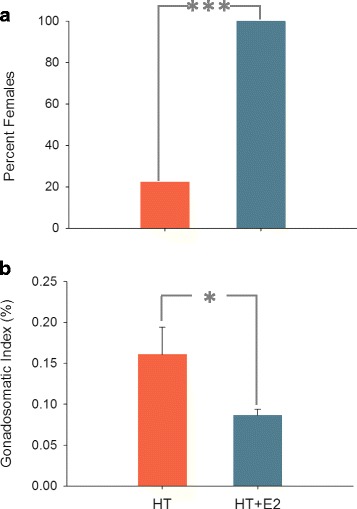
Effects of thermal and estrogen exposure on one-year-old European sea bass gonads. **a** Percent females in each group, with a difference from a Fisherian 1:1 balanced sex ratio. **b** Female gonadosomatic index. Data as mean + SEM. Males were not included since no males were present in the E_2_-treated group. * = *P* < 0.05; *** = *P* < 0.001

Sex ratio was female- (100% females) and male- biased (21% females) at the HT-E_2_ and HT groups, respectively (Fig. 1a) with statistically significant differences between them (*P* < 0.001) and between HT and LT, but not between HT-E_2_ and LT-E_2_ (*P* > 0.05, Additional file 6: Figure S1). At 337 dph, the GSI was significantly higher in HT females when compared to the HT-E_2_ females (*P* < 0.05) (Fig. 1b), but with no differences when compared to LT or LT-E_2_ (Additional file 6: Figure S1). Females were still immature, with ovaries replete with oocytes at the cortical alveolar stage (Additional file 7: Figure S2a-b). On the other hand, HT group males were fully mature and presented seminiferous tubules filled with sperm (Additional file 7: Figure S2c).

### Transcriptomic analysis of gene expression in sexually differentiating gonads

**Fig. 2:**
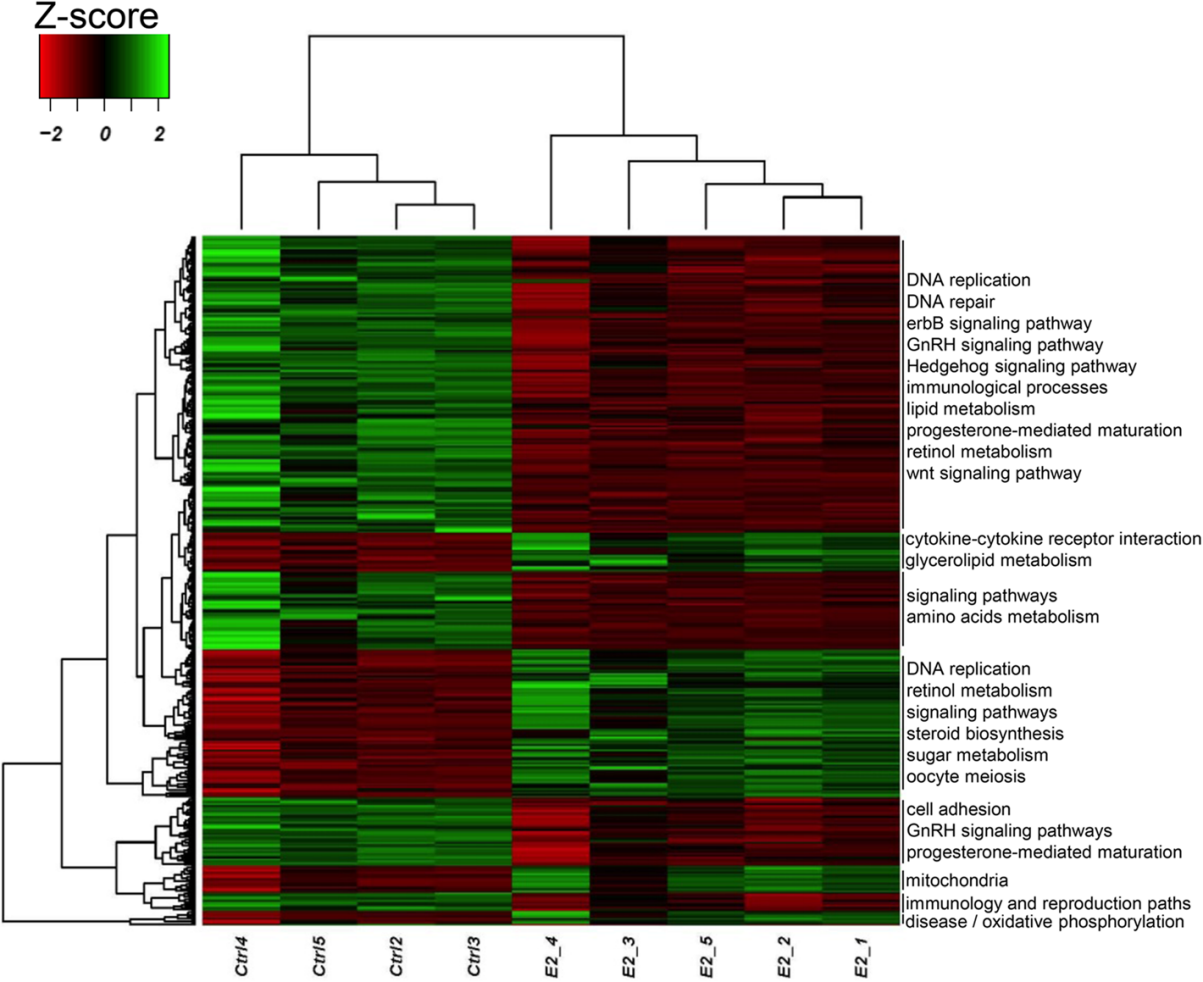
Individual heatmap representation of the differentially expressed genes in European sea bass 170 dph gonads from HT-E_2_ vs. HT group fish. Low to high expression is shown by a gradation color from red to green. On the right, the hierarchical clustering of genes by DAVID is shown (for a more tailed list of the altered pathways see Additional file 8: Table S3)

**Fig. 3:**
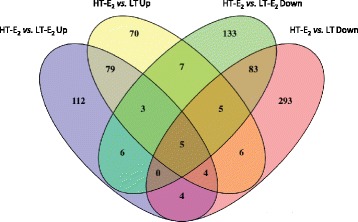
Venn diagram representation of the common up- and down-regulated genes for the comparisons HT-E_2_ vs. LT and HT-E_2_ vs. LT-E_2_)

Here we focus on the analysis of gonads undergoing different developmental pathways by using the masculinization effect of high temperature to force fish to develop as males and then either rescuing the phenotype by administering E_2_ (HT-E_2_ group) or studying the temperature-resistant females (HT group). A Principal Component Analysis showed how samples clustered on a treatment-manner (Additional file 5: Figure S4) and further microarray analysis yielded 383 significantly differentially expressed genes (DEG) where 92 were up- and 291 downregulated (Additional file 8: Table S3 and Additional file 9: Table S4). Hierarchical clustering based on the DEG showed that fish clustered in a treatment-related manner (Fig. 2). The associated pathways analyzed by DAVID (Additional file 9: Table S4), suggested that E_2_ exposure inhibited pathways processes related to DNA replication and repair, reproduction (progesterone-mediated oocyte maturation), hormonal-signaling (gonadotropin releasing hormone GnRH, epidermal growth factor erbB or Hedgehog), lipid metabolism or immunology. In contrast, pathways related to oocyte meiosis, steroid biosynthesis, sugar metabolism or cytokine receptor interactions were induced (Fig. 2, Additional file 10: Table S5). The comparison between LT (fish reared at low temperature allowing female development) and LT-E_2_ (fish reared at low temperature but forced to develop as females) had just 2 DEG, showing no differences between natural and artificial females (data not shown). On the other hand, when analyzing the common DEG between the double comparisons of the HT-E_2_ group vs. LT or vs. LT-E_2_ there were 91 upregulated and 93 downregulated common genes in the E_2_ group (Fig. 3, Additional file 11: Table S6). These genes were mainly involved in increased DNA repair, mitotic cell cycle and apoptosis; and in a reduction of the integration of energy metabolism, adherent and tight junctions, adipocytokine, epithelial cell signaling pathways and muscle contraction (data not shown).

**Fig. 4:**
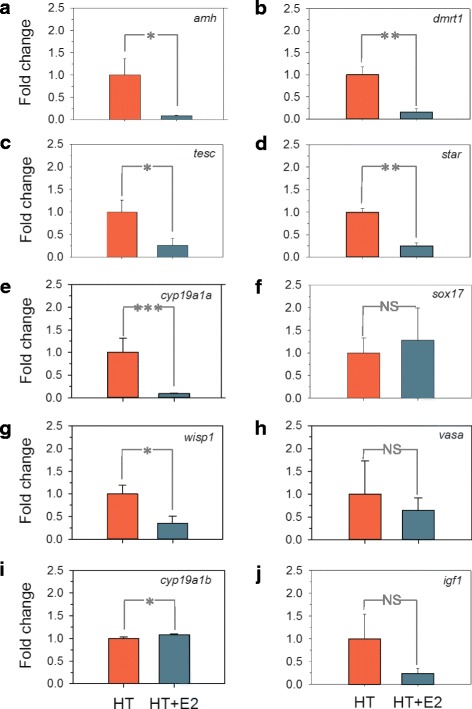
Quantitative real time PCR validation results for genes related to testis differentiation (**a**-**c**): anti-Müllerian hormone (*amh*), doublesex- and mab-3-related transcription factor 1 (*dmrt1*) and tescalcin (*tesc*); (**d**) cholesterol import: steroidogenic acute regulatory protein (*star*); ovarian differentiation (**e**-**h**): gonadal isoform of aromatase (*cyp19a1a*), SRY-related HMG-box transcription factor SOX17 (*sox17*), Wnt inducible signaling pathway protein 1 (*wisp1*), and vasa protein (*vasa*); (**i**) the neural isoform of aromatase (*cyp19a1b*), and (**j**) insulin-like growth factor 1 (*igf1*). Asterisks indicate significant statistical differences between groups (* = *P* < 0.05, ** = *P* < 0.01, *** = *P* < 0.001)

**Fig. 5:**
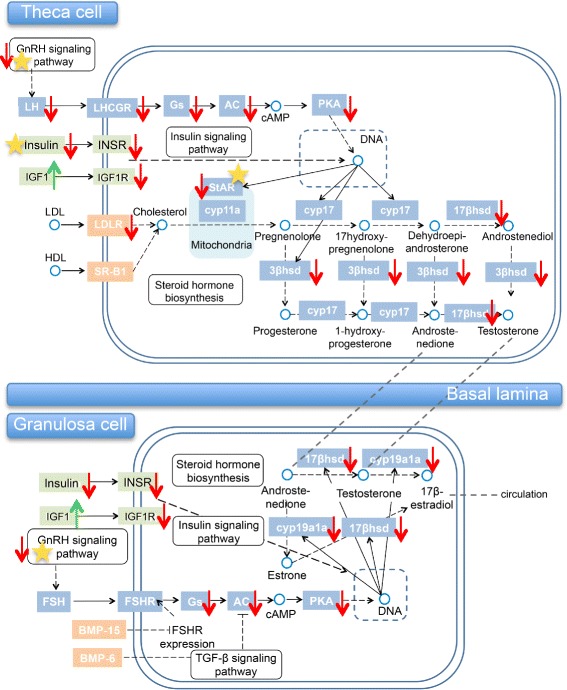
A KEGG pathway-based figure depicting the ovarian steroidogenesis pathway. Microarray results are marked with arrows (green arrows indicate a fold change (FC) higher than 1.5, while red arrows indicate a FC lower than 1.5). Yellow stars note genes in the microarray HT-E_2_ vs. HT comparison

### Validation of microarray results by qPCR

qPCR of some genes relevant for growth and reproduction were used to validate the array (Fig. 4 and Table 3). E_2_ exposure significantly (*P* < 0.05) downregulated genes related to testis differentiation, such as the anti-Müllerian hormone (*amh; P* < 0.05), doublesex- and mab-3-related transcription factor 1 (*dmrt1; P* < 0.01) and tescalcin (*tesc;P* < 0.05) (Fig. 4a-c, respectively). It also downregulated cholesterol import-related genes such as the steroidogenic acute regulatory protein (*star; P* < 0.01; Fig. 4d) and two ovarian differentiation-related genes: gonadal aromatase (*cyp19a1a*; Fig. 4e) and the Wnt1-inducible-signaling pathway protein1 (*wisp1*; Fig. 4g). However, other genes related to ovarian differentiation such as the transcription factor *sox17* (Fig. 4f) and a germ cell marker, *vasa* (Fig. 4h) were not affected. The neural isoform of aromatase (*cyp19a1b*) was significantly upregulated (*P* < 0.05) by E_2_ exposure (Fig. 4i), while insulin-like growth factor-1 (*igf1*) was not altered (Fig. 4j). For half of the analyzed genes, their expression was increased in the E_2_ group when comparing fold change changes (data not shown).

**Table 3:** Microarray validation by qPCR for the HT-E_2_ vs. HT comparison

Microarray			qPCR	
Gene symbol	log2 Fold change	Adjusted *P* -value	log2 Fold change	Adjusted *P*-value
*amh*	−2.48	0.037*	−3.18	0.011*
*aqp1*	2.16	0.349	0.70	0.094
*col18a*	−4.28	0.035*	−0.78	0.174
*cyp19a1a*	−1.03	0.781	−3.47	0.000***
*cyp19a1b*	−1.37	0.089	0.12	0.028*
*dmrt1*	−1.54	0.007**	−2.39	0.004**
*gnrh*	1.11	0.129	−1.83	0.051
*igf1*	5.95	0.035*	−1.73	0.149
*mettl22*	−1.31	0.121	−1.21	0.113
*prl*	−1.03	0.806	−1.94	0.018*
*sox17*	1.08	0.779	0.35	0.570
*star*	−1.87	0.051	−2.00	0.006**
*tesc*	−1.17	0.324	−1.88	0.017*
*vasa*	–	–	−0.64	0.721
*wisp1*	1.02	0.816	−1.51	0.045*

Note: Asterisks note statistical significant differences: * = *P* < 0.05; ** = *P* < 0.01; *** = *P* < 0.001. *N* 10 individuals analyzed per gene

### Gene ontology enrichment analysis of genes regulated by exposure to estradiol

Blast2GO analysis enabled the identification of the associated GO terms for the DEG and related then to biological process (BP), molecular function (MF) or cell component (CC) and always showed more downregulated GO terms at any given comparison. The main subcategories were related to reproduction, signaling, responses to stimulus, growth, immune system and developmental processes. Binding and catalytic activities were the most abundant among the MF subcategories (Additional file 12: Table S7). A two-tailed Fisher’s exact test with multiple testing corrections for FDR (*P*-value filter of 0.05) was performed to assess the over-representation of the functions related to the downregulated genes due to E_2_ exposure. Specifically, there were 148 over-represented functions when taking the microarray as background (Additional file 13: Table S8). The most interesting enriched MF GO-terms (26) were related to nuclear hormone receptor binding, growth factor and NADP-retinol hydrogenase activity. Enriched GO-terms (97) were clearly related to reproduction, immunology and growth.

### KEGG pathway enrichment analysis of genes regulated by estradiol exposure

There were 46 pathways affected by the E_2_ treatment (Additional file 14: Table S9), mainly related to metabolism (i.e. retinol), immunological signaling and steroid hormone biosynthesis. Furthermore, DAVID analysis on the GO-terms with the highest stringency showed that meiosis (two clusters with 1.47 and 1.06 enriched scores), reproduction (three clusters with 1.17, 0.81 and 0.4 enriched scores) and hormone regulation (one cluster with 0.33 enriched scores) were the most enriched ones. Since we found the genes involved in reproduction to be downregulated as a result of the E_2_ exposure, we further focused on the genes related to the ovarian steroidogenic pathway, which for most of them showed the same tendency towards downregulation. Among them, *star* and *gnrh* genes exhibited a significant downregulation (*P* < 0.05). On the other hand, *igf1* exhibited an opposite significant increase in the expression (*P* < 0.05) due to the E_2_ exposure (Fig. 5).

### Protein-protein interaction analysis

Proteins coded by the DEG analyzed using STRING, showed enrichment in interactions (*P <* 0.001). E_2_ exposure caused an increase in protein interactions (range of combined scores of interactions 0.400–0.999) for protein networks related to transcriptional activation, DNA repair, immunity, catabolism, oxidative phosphorylation and muscle contraction (Additional file 15: Figure S5). On the other hand, protein networks obtained from the downregulated genes (range of combined scores of interactions 0.402–0.999) were more related to: apoptosis, inflammation, histone demethylases and inhibition of histone acetylase 1, cell adhesion, morphology and motility, protein complex assembly, intracellular trafficking and secretion, Rho and Rac GTPases activators, response to hormonal stimulus and reproductive structure development (Additional file 16: Figure S6).

### Genes related to epigenetic regulatory mechanisms

We found that demethylases, *dicer1*, helicases, most of the histone deacetylases, polycomb complex members, as well as DNA-methyltransferases 1 and 3 were downregulated in the HT-E_2_ group. In contrast, most histone acetyltransferases and methyltransferases were upregulated. Finally, histone acetylase 11 (*hdac11*) and euchromatic histone lysine N-methyltransferase (*ehmt2*), two genes previously analyzed by qPCR, showed a heat-related upregulation even under the E_2_ exposure (Additional file 17: Table S10).

## Discussion

In this study, European sea bass, a fish with mixed genetic and environmental influences [40, 41], was used to analyze the transcriptome of fish gonads at the time of sex differentiation after being exposed to thermal and chemical perturbations. Here we show how the exposure to estrogen at early juvenile development was able to completely feminize the population that otherwise would have developed as males due to the high temperature. Moreover, estrogen-exposed females showed a transcriptome reprograming that affected not only steroidogenesis but also pathways related to reproduction, immunity, growth, response to stimulus and the metabolism of lipids and xenobiotics. However, based on histological analysis, we observed that once the female phenotype is imposed gonads could proceed with apparent normal development.

The HT group was masculinized (21% females) by the elevated temperatures. However, this masculinizing effect was completely overridden by the E_2_ exposure (100% females at HT-E_2_ group) without affecting the histological structure of the immature ovary, where cortical alveolar oocytes in both groups predominated. However, GSI values indicate a reduction in ovarian growth as already described in other fish species [64–66]. GSI values of E_2_-exposed females are higher than those of control females, without any apparent effect on fat content, opposite to what was found by Saillant et al. [14], and with slightly higher HSI in E_2_-exposed females, in agreement with previous studies on fish subjected to the effects of xenoestrogens [67–69].

Microarray analysis showed that E_2_ exposure caused an alteration in the expression of 383 genes from gonads at the time of sex differentiation. The most important of those effects are discussed below.

### Reproduction

It is well established that *cyp19a1a* gene expression and aromatase enzyme activity are necessary for normal ovarian differentiation and maintenance in all non-mammalian vertebrates including fishes [3]. E_2_ treatment caused a significant downregulation of *cyp19a1a* at 170 dph, as assessed both by analysis of microarray and qPCR data. This downregulation took place after the hormonal exposure finished, as has also been observed in rainbow trout and zebrafish [34, 70]. Nevertheless, since 1) E_2_ completely feminized the exposed fish, even after initial heat-induced masculinization, and 2) the European sea bass *cyp19a1a* promoter lacks estrogen response elements (EREs), as in other fish [71], these observations suggest that E_2_-induced feminization likely did not involve direct *cyp19a1a* regulation. This interpretation is supported by qPCR results showing that *star,* an upstream component of the steroidogenic pathway, also was significantly downregulated by E_2_, in agreement with previous results in zebrafish [70], suggesting that E_2_ shuts down the first steps of steroidogenesis by blocking *star* expression. Furthermore, the microarray analysis showed a whole downregulation of the ovarian steroidogenesis pathway and this seems to happen in a dose- and species-dependent manner. This is supported by the fact that E_2_ expression decreases in some species and increases in some others [38, 66, 70, 72]; and because other downstream genes such as *cyp19a1a* and *17β-hsd* are affected in females but not in males [70, 72, 73]. However, studies in our lab have shown how the *cyp19a1a* expression downregulation does not involve changes in its promoter DNA methylation since unexposed and feminized females by E_2_ showed no differences in the gonadal aromatase promoter methylation levels [43]. Other genes related to ovarian differentiation (i.e., *wisp1, cyp19a1a*, *17β-hsd* and *star*) as well as male-related genes (*amh*, *dmrt1* and *tesc*) or *cyp19a1b a*nd *sox17* were downregulated after E_2_ exposure, although in contrast to what has been found in other fish species [66].

### Immunity

Some studies have suggested the possibility that sex steroids affect the immune system [74]. The microarray we used has a good representation of immunity-related terms and contains probes for a group of genes constituting the signaling pathways responsible for generating the immune response, including the Toll-like, NOD-like, RIG-I-like and the T-cell receptor signaling pathways. Interestingly, the latter was downregulated in the E_2_ group. Also, many genes of the complement component, some cytokines and lysozymes were also downregulated after E_2_ exposure, as seen before for medaka [29, 30]. In contrast, several terms referring to response to stimulus were enriched, including response to estradiol, mechanical stimulus, lipopolysaccharids and regulation of response to stress.

### Xenobiotic metabolism

Microarray DEG showed three pathways related to drug and xenobiotic metabolism through cytochrome P450 downregulation. Furthermore, some proteins that are able to metabolize xenobiotics like the glutathione S-transferase proteins (GSTs; [75]) were up- (*gst* and *gstθ*) and downregulated (*gstα*, *gstk*, *gstm*) in the microarray. The latter is in agreement with what have been seen for *gstα* in goldfish when exposed to a hepatotoxin (*Carassius auratus*; [76]) or to *gstπ* in Atlantic salmon when exposed to tributyltin (*Salmo salar*; [77]).

### Growth

Sex steroids can influence fish growth by altering the GH-IGF system [78]. Furthermore, during the sexual maturation of some fish species [79–81], plasma levels of sex steroids and GH correlate indicating a crosstalk between reproduction and growth-related pathways. In this study, all genes related to growth hormone and its receptor were downregulated. The same occurred with the insulin-like growth factor II gene, its receptors, *igf1r,* and its associated binding proteins in opposition to what has been described for the fathead minnow [38, 66] when exposed only to E_2_. Further studies are needed since, at present, it is not possible to discern if these differences are species-specific or the result of the combination of both thermal and E_2_ exposures.

### Lipid metabolism

After E_2_ exposure, terms related to lipid metabolism such as white fat cell differentiation, regulation of fat cell differentiation, plasma lipoprotein clearance, apolipoprotein binding or high and low density lipoprotein particle remodeling were over represented. Similar downregulations of apolipoproteins have been shown in different fish species [82–84], but not in the mummichog (*Fundulus heteroclitus*; [39]). Moreover, *apoe,* a protein related to lipid uptake by oocytes, was also downregulated in our study, in agreement with what has been previously shown for zebrafish [85].

### Epigenetic regulatory mechanisms-related genes

Although it is known that epigenetic mechanisms are responsible for the acquisition and maintenance of cell identity, they have been only marginally explored in an ecological context. Thus, we have analyzed by qPCR the behavior of seven genes related to epigenetic regulatory mechanisms present in our microarray. Among them, *dicer1*, *jarid2a*, *pcgf2*, *suz12* and *mettl22* were downregulated by E_2_, overriding temperature effects. In contrast, the expression of *ehmt2* and *hdac11* was unchanged. Interestingly, we have also observed that the expression of six heat shock proteins (*hrsp12*, *hsbp1*, *hsp10*, *hsp60*, *hspa14* and *hsp70*) was upregulated, implying that early exposure to elevated temperatures had persistent effects on the gonadal transcriptome, effects that were not overridden by the subsequent E_2_ exposure.

## Conclusions

Taken together, these results show how at the population level all fish developed as females since estrogen exposure during early juvenile development is able to completely override the masculinizing effect of elevated temperatures. However, these fish developed as females despite showing at the time of sex differentiation a downregulation of key genes in steroidogenesis. This blockage happened not only at upstream genes of the pathway such as *star*, but also at downstream genes such as *cyp19a1a* and *17β-hsd*. Furthermore, estradiol administration also affected pathways related to reproduction, immunity, xenobiotic and lipid metabolism, signaling, responses to stimulus and growth. Thus, exposure to exogenous estrogens of sexually differentiating fish had a profound reprogramming effect on their gonadal transcriptome, causing not only a complete feminization of the population but an inhibition of steroidogenesis in developing females. It should be noted that some of the resulting females will be fish that otherwise would have developed as males. However, at 1 year of age, feminized fish exhibited a normal gonadal histology suggesting that once the female phenotype is imposed gonads can apparently continue their normal development. Although the impact of sex-reversed fish on natural populations has been simulated in species with simple chromosomal sex determining systems [86], the situation in species with more complex systems like the one used in this study remains unexplored. The data shown in this study helps to fill in the gap on the underlying mechanisms operating at the molecular level.

## Additional files

Additional file 1: Figure S3. Experimental design. Upper graph depicts thermal differences. High/low temperatures (21 °C/15 °C) are marked in red/orange and blue/purple, respectively. The duration of the Estradiol treatment (90 to 154 dph) is marked with a light orange box. The bottom panel marks the key events related to sex differentiation and maturation in the European sea bass, as well as the main sampling points. (TIFF 2702 kb)

Additional file 2: Figure S7. Two-step cluster analysis of 2DCt values to divide among high and low *cyp19a1a* expressors. a) LT (blue circles) and HT(red squares) *cyp19a1a* 2DCt values. b) LT-E2 (purple circles) and HT-E2 (orange squares) *cyp19a1a* 2DCt values. The black line denotes the median. (TIFF 2702 kb)

Additional file 3: Table S1. Gene abbreviation glossary of the genes analyzed by qPCR or selected from the list of differentially expressed genes. (XLSX 9.44 kb)

Additional file 4: Table S2. Quantitative PCR primer characteristics. (XLSX 11.3kb)

Additional file 5: Figure S4. Predicted protein-protein interactions by STRING for the 92 upregulated genes in the HT-E_2_ vs. HT comparison. (TIFF 2702 kb)

Additional file 6: Figure S1. Sex ratio and GSI. a) Female percent is shown in pink for all the four studied groups. b) GSI for females (pink) and males (blue) at 337 dph. Letters mark statistical differences between groups (females: uppercase; males: lowercase). (TIFF 2702 kb)

Additional file 7: Figure S2. Histological images of one-year-old European sea bass. a) HT females; b) HT-E_2_ females and; c) HT males. Scale bar = 50 μm. (TIFF 2702 kb)

Additional file 8: Table S3. Number of differentially expressed genes in 170 dph European sea bass gonads when comparing fishes from the HT-E_2_- vs. HT group during sex differentiation. (XLSX 9 kb)

Additional file 9: Table S4. Differentially expressed genes for the HT-E_2_ vs. HT comparison. (XLSX 29 kb)

Additional file 10: Table S5. Pathway analysis by DAVID of clusters of DEG for the HT-E_2_ vs. HT comparison. (XLSX 14 kb)

Additional file 11: Table S6. Common DEG when comparing HT-E_2_ (artificial) females vs. LT and LT-E_2_ (natural) females. (XLSX 18 kb)

Additional file 12: Table S7. GO results subdivided into three categories: biological process (BP), molecular function (MF) and cell component (CC) for the HT-E_2_ vs. HT comparison. (XLSX 12.3 kb)

Additional file 13: Table S8. Fisher’s exact test with multiple testing corrections of FDR results. (XLSX 17.1 kb)

Additional file 14: Table S9. KEGG pathways derived from the differentially expressed genes (DEG) that were either up- or downregulated in the HT-E_2_ vs. HT comparison. (XLSX 11.3 kb)

Additional file 15: Figure S5. Principal Component Analysis (PCA). Individuals from each group are marked with red squares (HT), orange squares (HT-E_2_), blue circles (LT) or purple circles (LT-E_2_). (TIFF 2702 kb)

Additional file 16: Figure S6. Predicted protein-protein interactions by STRING for the 291downregulated genes in the HT-E_2_ vs. HT comparison. (TIFF 2702 kb)

Additional file 17: Table S2. List of the genes related to epigenetic regulatory mechanisms discussed in the text for the HT-E_2_ vs. HT comparison. (XLSX 12 kb)

## Abbreviations

*amh*: Anti-Müllerian hormone
*aqp1*: Aquaporin 1
BP: Biological process
BW: Body weight
CC: Cell component
CI: Carcass index
*col18a1*: Collagen alpha-1 (XVIII) chain
*cyp19a1a*: Cytochrome P450, family 19, subfamily A, polypeptide 1a
*cyp19a1b*: Cytochrome P450, family 19, subfamily A, polypeptide 1b
DEG: Differentially expressed genes
*dicer1*: Endoribonuclease Dicer
*dmrt1*: Doublesex- and mab-3- related transcription factor I
dph: Days post hatch
E_2_: Estradiol-17ß
*ehmt2*: Euchromatic histone-lysine N-methyltransferase 2
erB: Epidermal growth factor receptor
EREs: Estrogen response elements
FC: Fold change
FDR: False discovery rate
GEO: Gene expression Omnibus
*gnrh*: Gonadotropin-releasing hormone
GSI: Gonadosomatic index
GSTs: Glutathione S-transferase proteins
*hdac11*: Histone deacetylase 11
HIS: Hepatosomatic index
hrsp12: Heat responsive protein 12
hsbp1: Heat shock binding protein
HSP: Hormone-sensitive period
hsp10: Heat shock protein 10
hsp60: Heat shock protein 60
hspa14: Heat shock protein 14
hspa70: Heat shock protein 70
HT: Control high temperature
HT-E_2_: Estradiol high temperature
*igf1*: Insulin-like growth factor I
*jarid2a*: Protein Jumonji
LT: Control low temperature
LT-E_2_: Estradiol low temperature
*mettl22*: Methyltransferase-like protein 22
MF: Molecular function
PCA: Principal component analysis
*pcgf2*: Polycomb group ring finger 2
*prl*: Prolactin
qPCR: Quantitative real-time PCR
*r18S*: r18S
SGD: Sexual growth dimorphism
SL: Standard length
*sox17*: HMG-box transcription factor SOX17
*star*: Steroidogenic acute regulatory protein
*suz12*: Suppressor of zeste 12 homolog
*tesc*: Tescalcin
*vasa*: Vasa protein
*wisp1*: WNT1 inducible signaling pathway protein 1
