# Supplementary figures and images for: Estrogen exposure overrides the masculinizing effect of elevated temperature by a downregulation of the key genes implicated in sexual differentiation in a fish with mixed genetic and environmental sex determination
Source: BMC Genomics. 2017 Dec 18;18:973. doi: 10.1186/s12864-017-4345-7 (PMC5735924; doi:10.1186/s12864-017-4345-7)

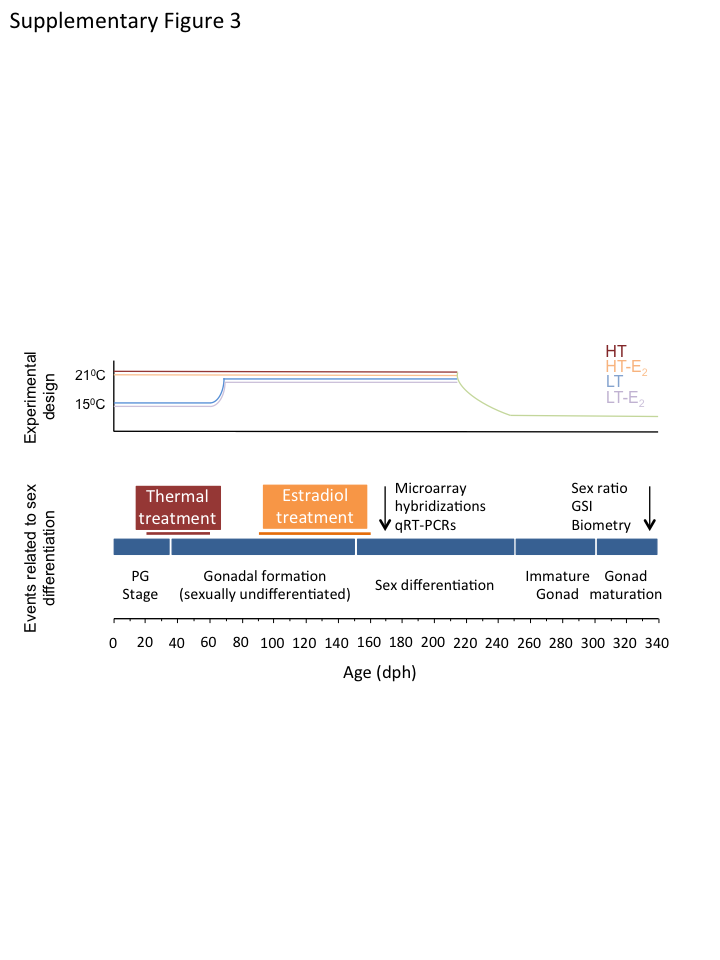

Supplement: Supplementary file 1 — Experimental design. Upper graph depicts thermal differences. High/low temperatures (21 °C/15 °C) are marked in red/orange and blue/purple, respectively. The duration of the Estradiol treatment (90 to 154 dph) is marked with a light orange box. The bottom panel marks the key events related to sex differentiation and maturation in the European sea bass, as well as the main sampling points. (TIFF 2702 kb) [file 12864_2017_4345_MOESM1_ESM.tif]

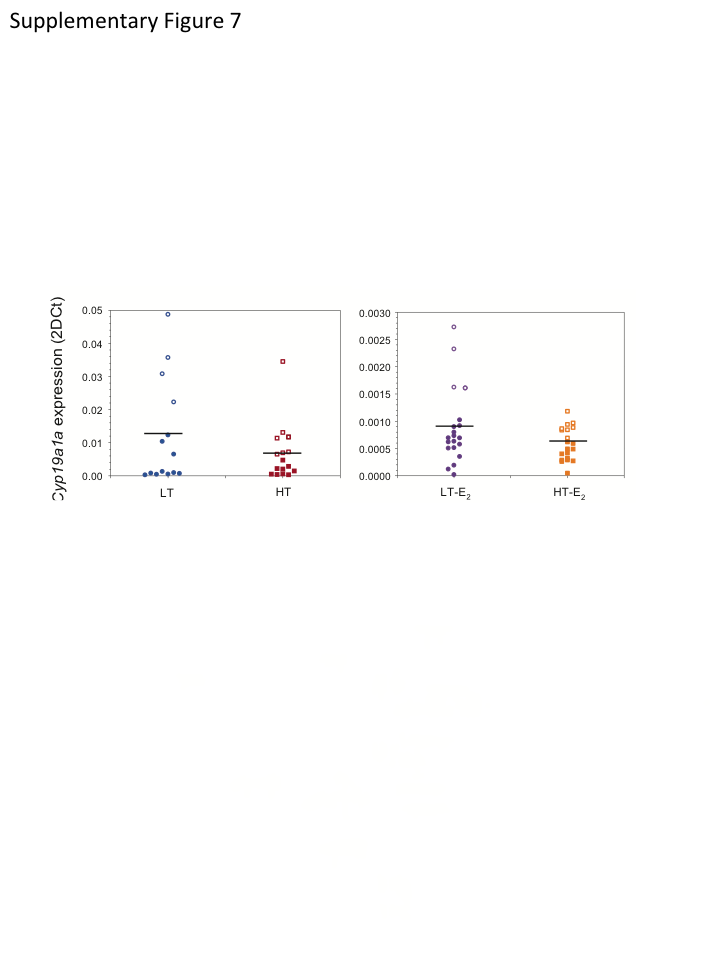

Supplement: Supplementary file 2 — Two-step cluster analysis of 2DCt values to divide among high and low cyp19a1a expressors. a) LT (blue circles) and HT(red squares) cyp19a1a 2DCt values. b) LT-E2 (purple circles) and HT-E2 (orange squares) cyp19a1a 2DCt values. The black line denotes the median. (TIFF 2702 kb) [file 12864_2017_4345_MOESM2_ESM.tif]

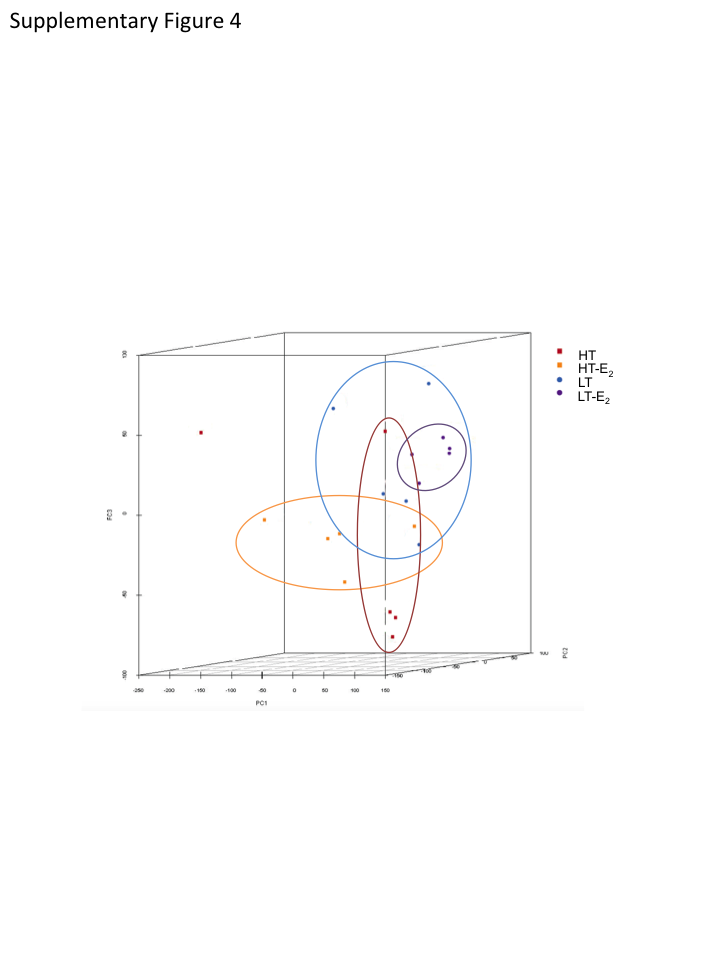

Supplement: Supplementary file 5 — Predicted protein-protein interactions by STRING for the 92 upregulated genes in the HT-E2 vs. HT comparison. (TIFF 2702 kb) [file 12864_2017_4345_MOESM5_ESM.tif]

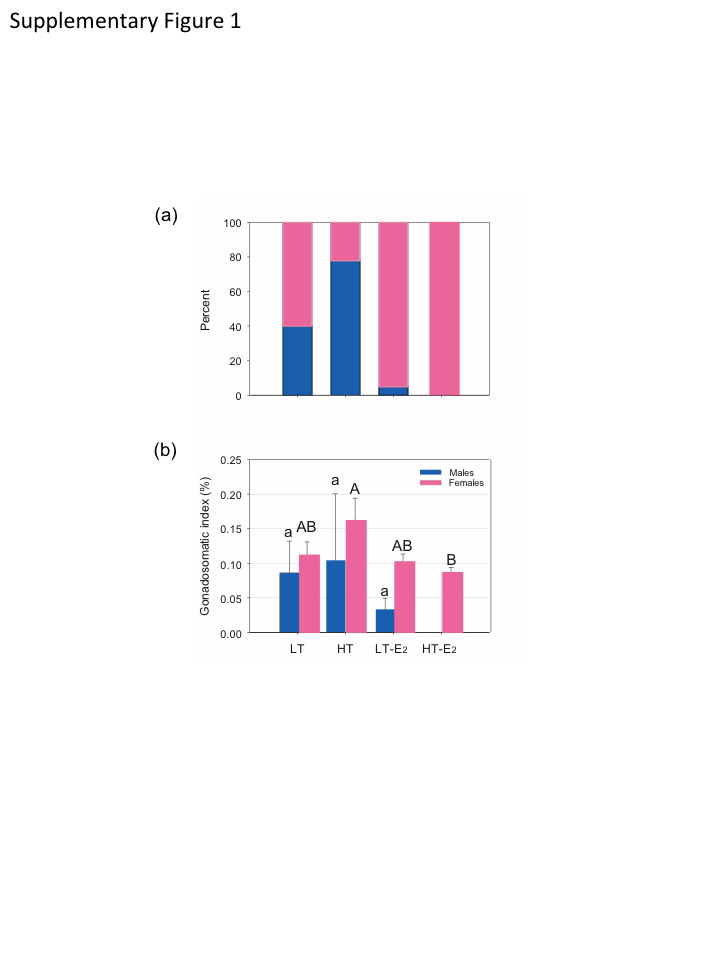

Supplement: Supplementary file 6 — Sex ratio and GSI. a) Female percent is shown in pink for all the four studied groups. b) GSI for females (pink) and males (blue) at 337 dph. Letters mark statistical differences between groups (females: uppercase; males: lowercase). (TIFF 2702 kb) [file 12864_2017_4345_MOESM6_ESM.tif]

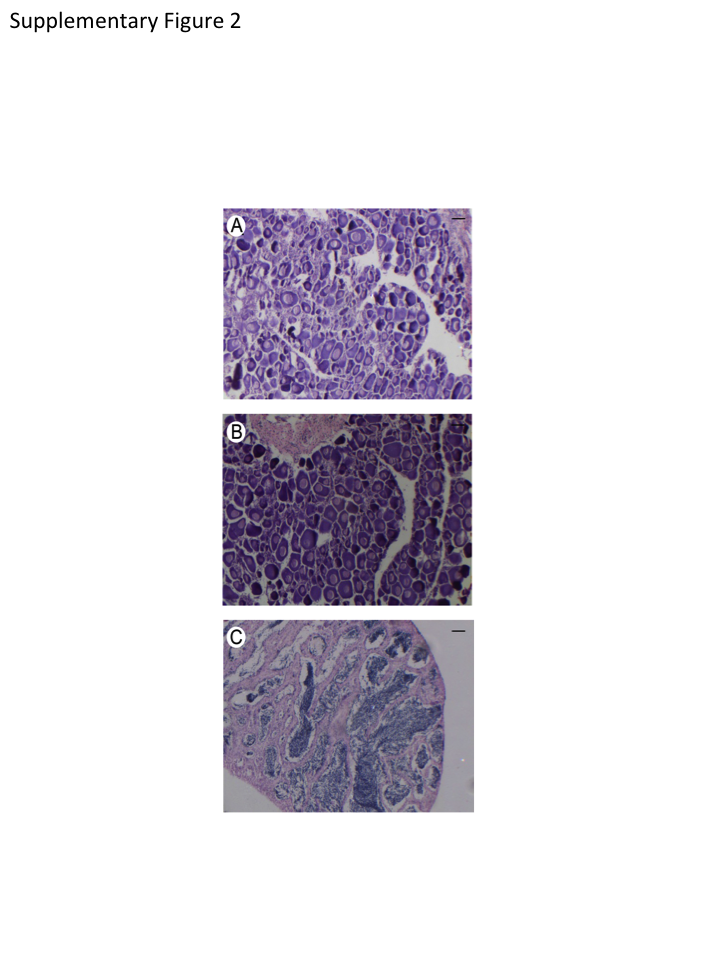

Supplement: Supplementary file 7 — Histological images of one-year-old European sea bass. a) HT females; b) HT-E2 females and; c) HT males. Scale bar = 50 μm. (TIFF 2702 kb) [file 12864_2017_4345_MOESM7_ESM.tif]

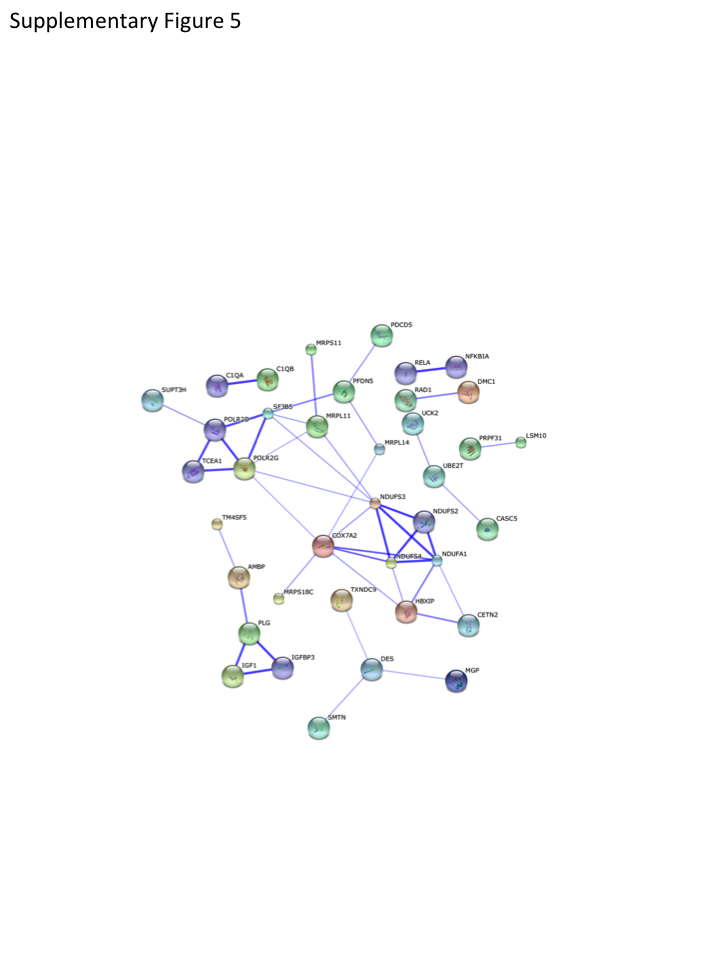

Supplement: Supplementary file 15 — Principal Component Analysis (PCA). Individuals from each group are marked with red squares (HT), orange squares (HT-E2), blue circles (LT) or purple circles (LT-E2). (TIFF 2702 kb) [file 12864_2017_4345_MOESM15_ESM.tif]

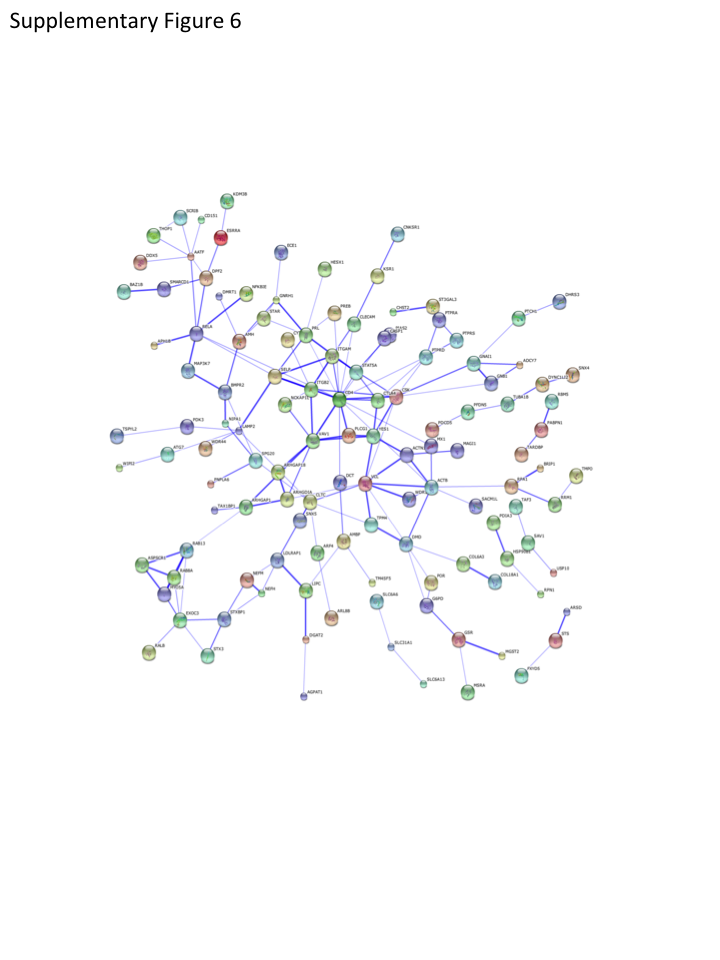

Supplement: Supplementary file 16 — Predicted protein-protein interactions by STRING for the 291downregulated genes in the HT-E2 vs. HT comparison. (TIFF 2702 kb) [file 12864_2017_4345_MOESM16_ESM.tif]
